# Cervical cancer in 2 women with a Mirena^®^-pitfalls in the assessment of irregular bleeding: a case series

**DOI:** 10.1186/1757-1626-1-62

**Published:** 2008-07-26

**Authors:** Sabina de Weerd, Pieter J Westenend, Sjarlot GS Kooi

**Affiliations:** 1 Department of Obstetrics & Gynaecology, Albert Schweitzer Hospital, Dordrecht, The Netherlands; 2 Laboratory for Pathology, Dordrecht, The Netherlands

## Abstract

**Introduction:**

The levonorgestrel-releasing intra-uterine device (Mirena^®^) is an effective, long term form of contraception that leads to a significant reduction of menstrual bleeding among majority of women. However, irregular bleeding is quite common in initial Mirena^® ^users and may thereby mask underlying cervical pathology.

**Case presentation:**

Two women with cervical cancer and a Mirena^® ^initially presented with irregular bleeding, posing a diagnostic pitfall which resulted in doctor and patient delay.

**Conclusion:**

Proper evaluation of irregular vaginal bleeding, including cervical cytology, should be a prerequisite among all women opting for a Mirena^® ^and must be repeated in case of persisting symptoms.

## Introduction

The Mirena^® ^(levonorgestrel-releasing intra-uterine device) has proven to be an effective, long term and reversible form of contraception that leads to a significant reduction (90%) of menstrual bleeding among the majority of women [1]. It is thereby also an alternative to hysterectomy and endometrial ablation for menorrhagia, including among perimenopausal women [2]. However, since irregular bleeding is quite common in initial Mirena^® ^users, this symptom may mask underlying cervical pathology as presented below.

## Case 1

A 40-year old nulligravida was referred to our outpatient clinic because of menorrhagia and irregular bleeding since 2 years, unresponsive to oral contraceptives. Her general practitioner obtained a Papanicolaou (Pap) smear at initial presentation of symptoms, which revealed no abnormalities. Inspection of the cervix showed no lesions, polyps or unusual bleeding. By vaginal ultrasound an intramural fibroid was seen in the fundus, measuring 4,5 cm × 4,5 cm and no further uterine anomalies. The next day, a Mirena^® ^was inserted. She returned to our clinic 2 weeks later due to abdominal cramping. The Mirena^® ^was in place. She was treated with antibiotics and painkillers on a presumptive diagnosis of pelvic inflammatory disease. Two months later at follow-up, the Mirena^® ^was still in place and irregular bleeding continued. As this is a common symptom in the first 3 months of Mirena^® ^use, she was reassured and instructed to make a new appointment if the bleeding were to persist.

Seven months after initial referral, she was seen for a cyst of Bartholin. During follow-up of the cyst, inspection by speculum revealed inflammation of the cervix. A Pap smear was performed, and during colposcopic examination, a lesion suspicious for neoplasia with abnormal vessels was seen. Two biopsies were taken and revealed a moderately differentiated squamous cell carcinoma. The Pap smear was reported as squamous cell carcinoma. Revision of the initial smear showed that due to blood staining it was difficult to interpret, but in retrospect it was suspect of malignancy. Clinical staging allocated our patient to cervical cancer stage IB. She was referred to a centre for Oncology for a radical hysterectomy with pelvic node dissection.

## Case 2

A 29-year-old woman was referred by her general practitioner 2 months after delivery of her first child for insertion of a Mirena^®^. Her medical history revealed no prior gynaecological problems and she had no history of abnormal cervical cytology. Examination by speculum and ultrasound prior to insertion of the Mirena^® ^revealed no abnormalities. Due to ongoing irregular bleeding and abdominal pains since insertion, the Mirena^® ^was removed several months later and oral contraception was prescribed. The irregular bleeding was attributed to the Mirena^® ^and no Pap smear was obtained in this period. Nine months after initial referral, she was referred again by her general practitioner due to persisting irregular bleeding and rebound tenderness during bimanual palpation. An ulcerative lesion on the cervix was seen by speculum, suspect for malignancy. Cervical cytology revealed moderate dysplasia, biopsies a micro-invasive squamous cell carcinoma of the cervix. Clinical staging allocated her to stage IIA cervical cancer. Assessment of new biopsies revealed adenosquamous carcinoma. She was also referred to a centre for Oncology for a radical hysterectomy with pelvic node dissection. During surgery, however, the procedure was aborted due to tumour extension into the vesicouterine fold. Lymph nodes were negative and she was treated by radiotherapy with brachytherapy.

## Discussion

The cases presented above both illustrate the doctor's delay caused by treating young women presenting with vaginal bleeding with a Mirena^® ^without proper evaluation of its cause. Persistence of symptoms was attributed to the Mirena^®^. Results of prior long-term studies on cervical cytology in women using a Mirena^® ^have shown that there is no increased incidence of cervical intraepithelial neoplasia [3,4]. Nevertheless, delayed diagnosis of cervical neoplasia among women with a Mirena^® ^may be more common than presumed, considering the fact that irregular bleeding is almost inherent to initial Mirena^® ^use.

The second pitfall is the limited sensitivity of a Pap smear, varying from 50–80% depending on the study and type of cytology assessment used [5,6]. Among the 80 women diagnosed with cervical cancer at our clinic between 1999 and 2004, the first patient presented above was the only one with a screening history showing no abnormal cytology and on revision a Pap smear suspect for malignancy (unpublished data). Similar results of a misinterpreted Pap test have been published by others [7-10]. It may seem common sense to scrutinise a normal result if our clinical findings do not correlate, but it becomes more complicated if no evident cervical anomalies are seen. Furthermore, as our first case illustrates, a Pap smear should be repeated regardless of prior findings if new symptoms develop or irregular bleeding persists. It is therefore questionable whether it was correct to attribute the ongoing irregular bleeding to the fibroid alone since no new cervical cytology was obtained at time of initial referral.

In conclusion, we suggest that proper evaluation of irregular bleeding including cervical cytology should be performed among all women opting for a Mirena^®^, and must be repeated in case of persisting symptoms.

## Consent

Written informed consent was obtained from the patients for publication of this case report. A copy of the written consent is available for review by the Editor-in-Chief of this journal.

## Competing interest statement

The authors declare that they have no competing interests.

## Authors' contributions

SdW was responsible for interpretation of data and drafting of the manuscript, PJW analyzed the Pap smears of the cases presented and revised and interpreted the Pap smears of the 80 women diagnosed with cervical cancer at our clinic between 1999 and 2004 as described in the discussion, and GSK was responsible for acquisition of data and conception of the manuscript.
